# Vaccination homophily in ego contact networks during the COVID-19 pandemic

**DOI:** 10.1038/s41598-024-65986-2

**Published:** 2024-07-05

**Authors:** Ádám Stefkovics, Fruzsina Albert, Anna Sára Ligeti, Beáta Dávid, Szilvia Rudas, Júlia Koltai

**Affiliations:** 1 National Laboratory for Health Security, HUN-REN Centre for Social Sciences, Budapest, Hungary; 2 IQSS, Harvard University, Cambridge, MA USA; 3 Institute for Sociology, HUN-REN Centre for Social Sciences, Budapest, Hungary; 4 Institute of Mental Health, Semmelweis University, Budapest, Hungary; 5 Department of Social Research Methodology, Faculty of Social Sciences, Eötvös Loránd University, Budapest, Hungary

**Keywords:** Ego networks, Contact diary, Homophily, Vaccine hesitancy, COVID-19, Infectious diseases, Epidemiology

## Abstract

Vaccine hesitancy is an inevitable risk for societies as it contributes to outbreaks of diseases. Prior research suggests that vaccination decisions of individuals tend to spread within social networks, resulting in a tendency to vaccination homophily. The clustering of individuals resistant to vaccination can substantially make the threshold necessary to achieve herd immunity harder to reach. In this study, we examined the extent of vaccination homophily among social contacts and its association with vaccine uptake during the COVID-19 pandemic in Hungary using a contact diary approach in two cross-sectional surveys. The results indicate strong clustering among both vaccinated and unvaccinated groups. The most powerful predictor of vaccine uptake was the perceived vaccination rate within the egos’ social contact network. Vaccination homophily and the role of the interpersonal contact network in vaccine uptake were particularly pronounced in the networks of close relationships, including family, kinship, and strong social ties of the ego. Our findings have important implications for understanding COVID-19 spread dynamics by showing that the strong clustering of unvaccinated individuals posed a great risk in preventing the spread of the disease.

## Main

Homophily, a well-observed attribute of social networks refers to the tendency of social contacts to be similar to one another^1,2^. This similarity applies not only to socio-demographic characteristics such as gender^3^, educational attainment or race and ethnicity^4,5^, but also to attitudes, values, personality traits and behavioral aspects, including health behavior^6–8^. Furthermore, recent developments in information technology (e.g. social network sites) seem to amplify homophily and are tied to the emergence of echo chambers^9–11^.

Although homophily is usually ascribed as individuals interacting with those who share similar socio-cultural characteristics, thus forming social ties based on matching individual traits (*social selection*)^1^, it is difficult to disentangle from other contributing social processes, such as *social contagion*, when an individual’s behavior influences others within their social network to adopt similar behaviors^12–15^. The rationale behind social selection lies in its ability to facilitate smooth interactions with others, making them more rewarding^1,16–18^, and thus enhancing the feeling of being understood. Conversely, it is more challenging to sustain relationships with those who are dissimilar, often resulting in an increased loss of such ties^19^. Similarity is partly a result of structural constraints, whereby one can only maintain ties from a given pool of other people. Furthermore, the social structure of public spaces, which provide a social context for networks, generally fosters interaction with similar others^1,20–24^ and limits the opportunities for contact with dissimilar ones^22,25,26^. By contrast, the social contagion approach emphasises how peer-to-peer interactions lead to the adoption of similar behaviors amongst individuals in a social network^27^.

Whilst homophily is common, its extent can vary depending on the network type and in different social groups. Relationships are often classified based on their strength to be strong versus weak. Strength is a multidimensional concept and can be interpreted using the frequency of interaction, time spent together, level of trust/intimacy, how long ago the people got acquainted with each other, the quantity/diversity of resources provided/received^28,29^. Strong ties are typically close, intimate relations in the inner circles of people, such as partners, children, parents, siblings, close friends. Homophily appears to be robust in various relationship types, with a tendency to become stronger in multiplex ties^1^. The close and strong ties of the ego are especially characterized by multiplexity^30^. The level of homophily may also differ in various social groups based on age, gender, educational attainment or social class, with the tendency to be more characteristic at the ends of the social scales^31^.

As with other attitudes and behaviors, social networks serve as a primary channel for the spread of health-related behavior^6,13,27^. Homophily increases health inequalities by reducing the adoption of health innovations, including public health measures^1,6,32–34^: as less healthy individuals have fewer ties to healthy individuals, they are less exposed to their influence^1,33,35–37^, which further reduces the likelihood of their adoption of innovations^37,38^.

This phenomenon is also evident in the context of decisions regarding vaccination. Vaccination decisions tend to spread within social networks, which can eventually lead to vaccination homophily and vaccination clustering^27,39–44^. Vaccination homophily refers to the assortative mixing patterns concerning vaccination status^45^ and can be attributed to multiple determinants, such as the presence of confounding factors, like age^46^, geographic location^47^, or socio-economic status^48^, which are predictors widely correlating with factors shaping interaction patterns in society^49,50^. Family members, friends, or colleagues can encourage or discourage vaccination even by directly telling people what to do, as the theory of planned behavior^51^ suggests. Social networks can also amplify the impact of policies designed to promote vaccination^52^.

The observed homophilic patterns of vaccine hesitancy, in general, are also applied to the COVID-19 pandemic, which nevertheless created an unprecedented situation in several respects. The widespread impact of the pandemic, alongside the extensive international effort to rapidly develop and distribute a vaccine, has created a unique situation in which COVID-19 vaccine hesitancy has been a subject of intense global interest^53^. COVID-19-related vaccination decisions depend on various factors^54,55^. Socio-demographic factors^53,55–57^, institutional trust^58^, attitudes^59,60^, religiosity, political ideology, media environment-related factors, and previous vaccination history^55,59,61^ can all affect one’s decision. Although the importance of interpersonal factors, such as having anyone close directly affected by COVID-19^62^, or knowing someone who died of COVID-19^63^ has been given some attention, the potential role of interpersonal networks has been limited (for exceptions, see^64–66^. Hancean et al.^64^ demonstrated that people with similar opinions about COVID-19 vaccination tend to cluster together in personal networks. Furthermore, Are et al.^67^ have found a higher level of observed COVID-19-related vaccination homophily among household contacts compared to non-household contacts, and vaccine homophily seems to decrease as social network size increases.

Strong interconnectedness in unvaccinated populations may affect spreading dynamics by seriously threatening the possibility of reaching the threshold necessary to achieve herd immunity^45^. Thus, understanding the clustering of vaccination behavior during pandemics is a crucial task. This paper aims to analyze the role of vaccination homophily in egocentric networks on vaccination decisions during the COVID-19 pandemic and (1) provide insights regarding the characteristics of COVID-19-vaccination-related homophily, (2) examine the variability of COVID-19-vaccination-related homophily in various relational groups (e.g. household members, friends, etc.) and (3) analyze how vaccination patterns in the ego’s network are related to the ego’s vaccination decisions. Our study is the first to present evidence about vaccination homophily during the COVID-19 pandemic on a nationally representative sample using the contact-diary approach^68^.

## Results

The data analyzed in this paper is part of a larger data collection effort conducted in Hungary during the pandemic between April 2020 and June 2022. The goal was to monitor the behavior and attitudes of Hungarian society as a response to epidemic measures. During this period, in each month a cross-sectional survey was conducted using CATI methodology. The sample sizes of the monthly surveys varied between 1000 and 1500 and all surveys were representative of the Hungarian adult population by gender, age, education and type of settlement^69^. For the current analysis, we used a merged dataset of two such cross-sectional and nationally representative surveys from November 2021 and from June 2022 (N=2000). In both months the dynamic of vaccination in the adult population was quite constant meaning that the vaccination rate of the adult population approximately reached its plateau until the time of the data collections^70^. In these data collections, in addition to the general survey questions, we applied a contact-diary approach^68^(see the Methods section for more details) for exploring all the active face-to-face contacts of the respondents contacted on the previous day of the data collection^71^. Additionally, multiple types of information (e.g., their educational level, financial situation, age, etc.) were collected about these alters including vaccination status. This allowed us to calculate vaccination rates in the ego network of each individual.

### Vaccination rates and homophily in different networks

The average vaccination rate in the ego-centric contact network of the respondents was 83.4% which is consistent with the vaccination rate of the egos (83.0%). We found small variations in the average vaccination rates in the respondents’ ego networks when comparing the different types of contact networks (for detailed descriptive statistics see Table S2 in the Supplementary Information).

**Figure 1:**
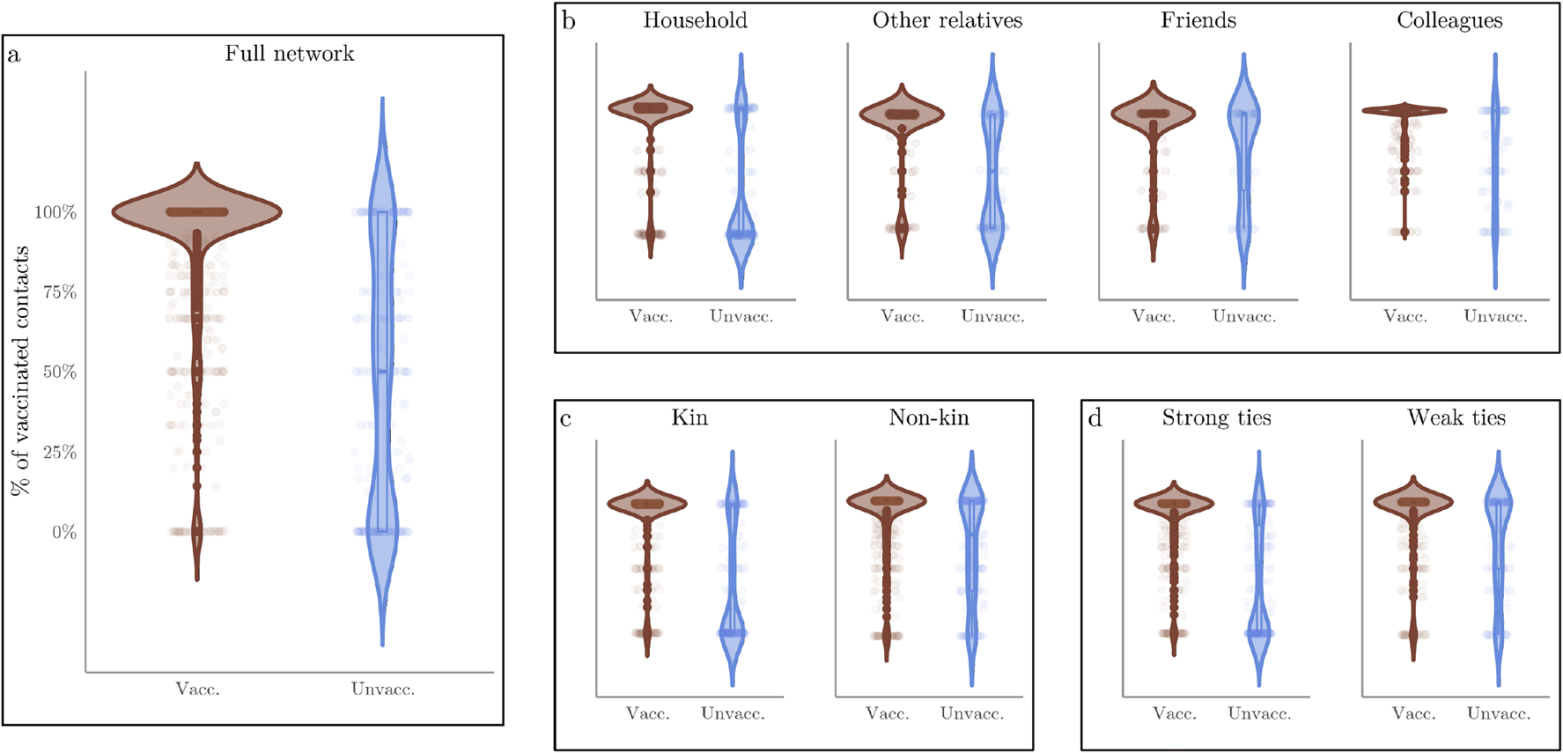
Distributions of vaccination rate in the different ego-networks of vaccinated and unvaccinated individuals.

Vaccination rates, however, differed strongly between contact networks of vaccinated and unvaccinated respondents. As shown in Fig. 1 and Table S3 in the Supplementary Information, the average vaccination rate in the full ego-centric network of vaccinated respondents was 90.5% compared to 48.7% observed among unvaccinated respondents. The median vaccination rate when comparing the full contact network of the vaccinated and the unvaccinated was 100% versus 50%. This difference is amplified within the household-, kin-, and strong tie networks. For instance, the average vaccination rates in the households of vaccinated versus unvaccinated respondents were 92.2% versus 30.5%, and the median vaccination rates were 100% versus 0%. As we widen the focus from household members to non-household member relatives, non-kin individuals, such as friends, and colleagues, or weak ties, differences between the networks of the vaccinated and the unvaccinated respondents slightly decrease, but remain high (88% vs. 57% on average).

**Figure 2:**
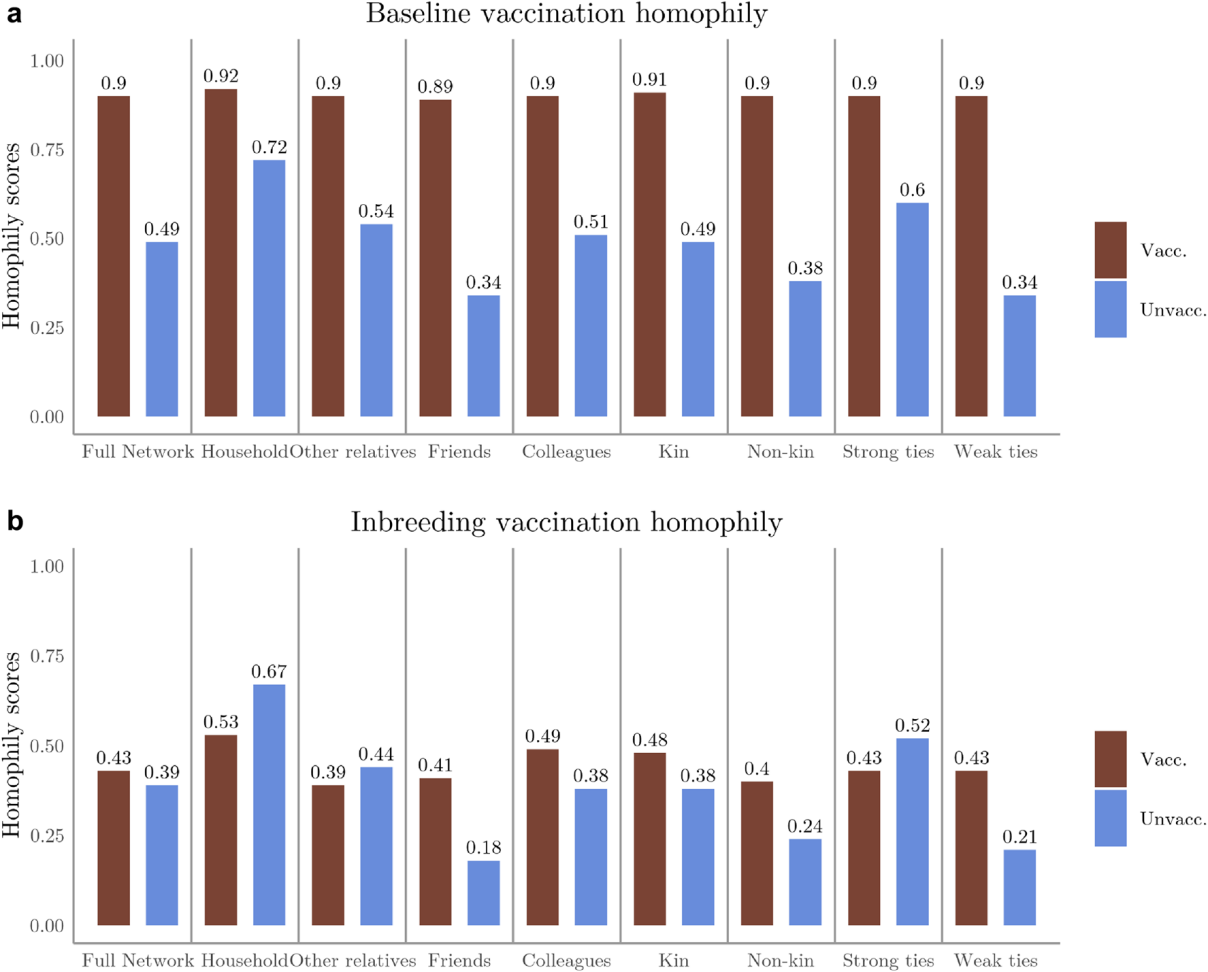
Baseline (**a**) and inbreeding homophily (**b**) scores in different ego networks of vaccinated and unvaccinated egos.

To discover the level of similarity in the vaccination status of the egos (respondents) and their alters, we calculated both baseline and inbreeding homophily scores^72,73^ in different types of contact networks of vaccinated and unvaccinated individuals separately (see Methods for more details). Results are presented in Fig. 2. Baseline homophily scores reflect the proportion of individuals in the ego’s networks who share the vaccination status of the ego. In all types of ego networks, baseline homophily scores were higher (0.89–0.92) among vaccinated egos compared to unvaccinated egos (0.39–0.72) in all types of networks. However, these results are partly affected by the fact that the proportion of vaccinated individuals in the sample is significantly higher (0.84) than the proportion of the unvaccinated group (0.17). Thus, to quantify how the ego networks are biased towards the vaccination status of the ego beyond the observed proportion of vaccination status in the sample, we also calculated the level of inbreeding homophily within the different contact networks. Positive inbreeding homophily scores indicate that homophily is more frequent than what would be anticipated by chance, whilst negative scores indicate heterophily^1^. Inbreeding homophily scores are consistently positive in both groups, especially regarding the closest (household, kin, strong tie) networks. It is somewhat higher in the complete contact network of the vaccinated individuals (0.43) compared to the unvaccinated ones (0.39), however, unvaccinated individuals have a stronger tendency for homophily in their household- (0.67 vs. 0.53), other kin- (0.44 vs. 0.39), and strong tie (0.52 vs. 0.43) networks than vaccinated individuals. In turn, vaccinated individuals have more homophilic friend-, colleague-, and weak tie contact networks in terms of vaccination status.

### The role of the contact network in vaccine uptake

To understand what role the interpersonal contact network of the respondents plays in the vaccination decision, we regressed multiple characteristics of the ego network along with the characteristics of the respondent on the vaccination status of the respondent, with a special focus on the network vaccination rate. Hierarchical binary logistic models were used, where network vaccination rate was added as a single predictor in Model 1. In Model 2 other network characteristics were included (e.g., network size, network composition), and finally, in Model 3, we added individual characteristics as controls, such as the respondent’s socio-demographic characteristics. Among the individual characteristics, we also included selective social media exposure, as according to the above mentioned earlier results^9–11^, it can amplify homophily and lead to echo chambers. The variable measured if the respondent used *only* social media as a news source about COVID-19-related issues (see the Methods section). The hierarchical structure of the models allowed us to capture the explanatory power of these different sets of predictors.

**Figure 3:**
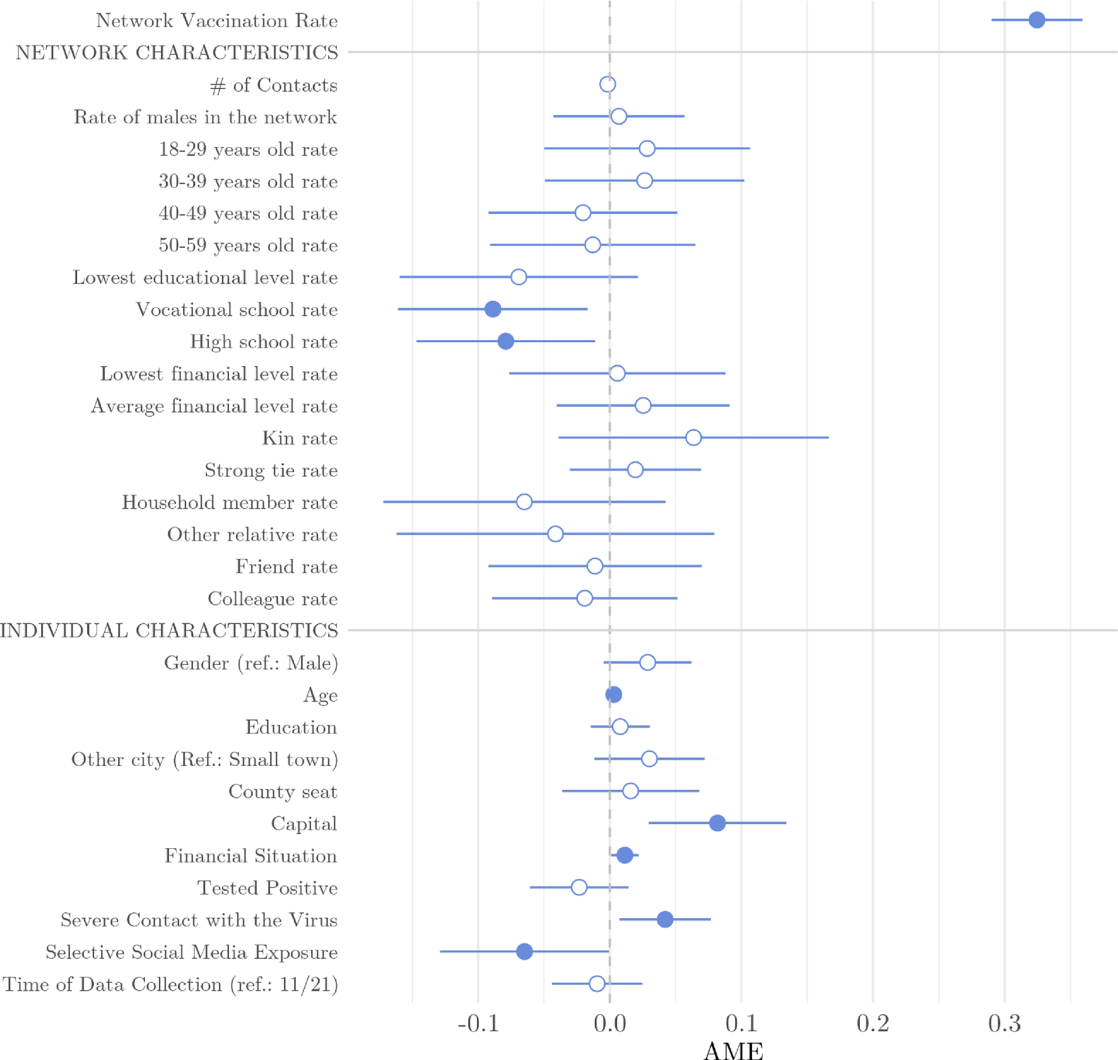
Average marginal effects of the final model fitted on vaccine uptake of the ego. Illustration of the results of Model 3 (see Table S4 in the Supplementary Information for detailed results.) Blue dots denote statistically significant associations at a 5% level. Nagelkerke’s $R^2$=0.30.

The Pseduo R-squared value (0.25) of Model 1 shows that the interpersonal network’s vaccination rate itself strongly predicts the ego’s vaccination status (see Table S4 in the Supplementary Information). In contrast, both network and individual characteristics have minimal additional impact on the dependent variable (increase in Pseudo R-squared: 0.02 and 0.034). In line with this, in Model 3, where all other network characteristics and control variables are included, the comparison of the average marginal effects (AMEs) shows that the vaccination rate of the full network was by far the strongest predictor of vaccine uptake (AME=0.32, SE=0.02, see Fig. 3). Besides, higher rates of individuals with vocational or high school degrees in the network decreased the probability of being vaccinated. The probability of being vaccinated was lower in smaller towns and cities and among those with selective social media exposure. Contrarily, vaccine uptake was positively associated with age, better financial situation, and if the respondent or their close individual has had severe experience with the virus. Other independent variables present in the model were not associated with vaccine uptake.

**Figure 4:**
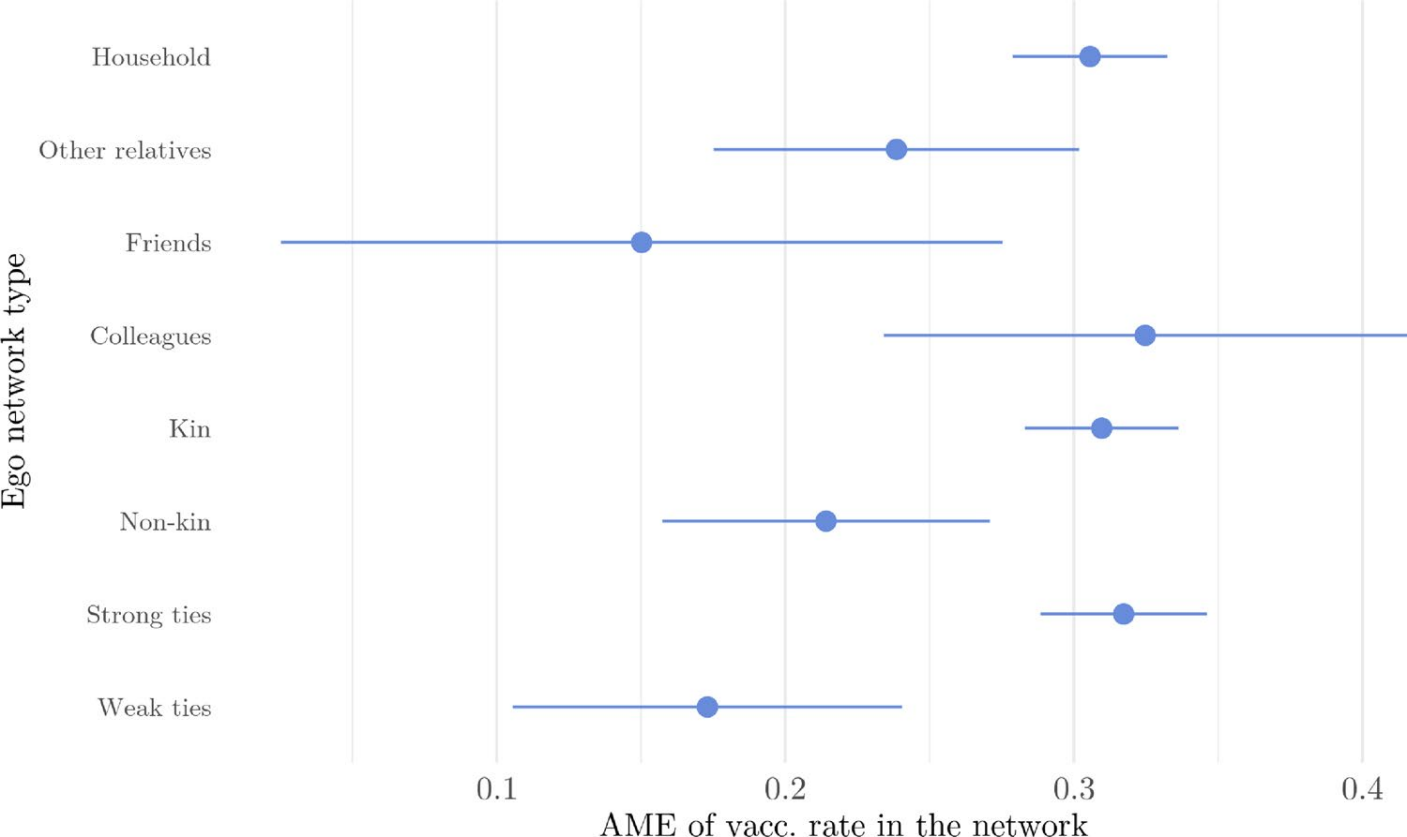
Average marginal effects of vaccination rates on vaccine uptake of the ego in different sub-networks of the ego. Illustration of the results of Model 3 (see Table S5, S6 in the Supplementary Information for detailed results.) Illustrated results are controlled for network- and individual-level characteristics.

Finally, models with the same structure as Model 3 were fitted in each sub-network of the ego. These analyses help to understand which networks matter the most in the vaccine uptake of the ego. As shown in Fig. 4 and Table S5-6, the association between the network vaccination rate and vaccine uptake of the respondent is the strongest in household-, colleague-, kin-, and strong tie networks, and the weakest in the friend network.

## Discussion

Homophily is a significant factor that shapes the required vaccine coverage for achieving herd immunity^45^. Strong clustering among hesitant individuals can make the goal of herd immunity difficult or nearly impossible to achieve. This study assessed vaccination homophily and its association with vaccine uptake during the COVID-19 pandemic in Hungary. We found strong clustering among both vaccinated and unvaccinated individuals. Homophily was especially strong in household-, kin-, and strong tie networks of the ego. Moreover, the vaccination rate of the network was by far the single strongest predictor of the ego’s vaccine uptake.

Our findings regarding the strong clustering in COVID-19 vaccination decisions corroborate previous reports^64,66,67^, and extend our understanding of the determinants of vaccine hesitancy. Two explanations emerge for these assortative patterns: vaccination decisions can be the result of influence-based contagion of behavior or they can emerge by shared socio-demographic traits through the process of social selection^74^. Furthermore, social selection may not only occur along socio-demographic traits but people may also choose to socialize with others who share their vaccination status. While this study was not specifically tailored to assess the causes of vaccination homophily, the evidence we present seems to hint at the influence of social contagion. In our models, we controlled for a range of socio-demographic characteristics of both the ego and the ego’s networks, which, to some extent, mitigate the likelihood of selection influences playing a role. Nevertheless, future longitudinal or qualitative studies are needed for a more comprehensive understanding of the roots of vaccination homophily. The results of these studies would greatly help policy interventions as each underlying cause requires a distinct approach^12,74^.

We fine-grained the results of previous research by showing that the degree of vaccination homophily is a function of the type of the ego-network. Vaccination homophily was more common in tight-knit communities which resonates well with the observation that people often gravitate towards others similar to themselves and are less similar to those more distant in social circles^67^. Our findings contradict McPherson et al.^1^ claim that strong, affective and slowly decaying family ties often allow for greater heterophily in terms of attitudes and behavior. In our study, these relationships formed the bedrock for homophily. This aligns with the observation that closer relationships often harbor greater trust and serve and act as significant channels for the propagation of social norms and behavior^66,75^. In contrast to some earlier approaches that categorized families and friends together in one group^40,42,43,76^, our evidence suggests that a more specific network-type characterisation is essential when examining vaccination homophily. One exception was colleagues. A possible explanation for the strong clustering in vaccination decisions in workplaces can be that in Hungary, COVID-19 vaccination was mandated by law in certain professions (e.g. healthcare workers), and many companies required their employees to get vaccinated, which increased the level of COVID-19 vaccination homophily at those workplaces.

This study offers some important implications for understanding COVID-19 spread dynamics. The assortative patterns suggest that unvaccinated individuals posed a much greater risk to other unvaccinated people, and their impact on vaccinated individuals may have been limited as they contacted one another less frequently. Consequently, due to the interconnectivity of the unvaccinated population, it may remain a challenge to reach the threshold needed for herd immunity, even when vaccination rates are high in society. The increased risk in these communities can lead to localized outbreaks or “hotspots” of infection. This underscores the importance of tailored preventive measures and public health messaging^74^. Communication strategies tackling vaccine hesitancy in the later waves of the pandemic need to consider the unique concerns, questions, and informational needs of unvaccinated, partially vaccinated and vaccinated groups separately. Furthermore, given that vaccine hesitancy tends to cluster socially (in tight-knit communities) and geographically, community-centred and culturally aware prevention strategies are advised^74^.

Our study comes with some limitations, which offer opportunities for future research. First, information about the contacts was collected from the respondents which likely introduced some measurement error. For instance, it is possible that in some cases the information reported by the respondents was inaccurate. Bias can be present because respondents did not remember all their contacts due to recall bias^77^, as it can also happen that respondents may have overreported vaccination rates among their contacts because of social desirability bias^78^, or they might have mistakenly perceived that their contacts were more like them than they truly were (false consensus effect)^79^. However, perceived social norms influence health behavior, such as beliefs about other people’s behavior. Agranov et al.^80^ found a strong link between perceived vaccination social norms and vaccination intentions. Thus, even if a certain contact is only believed to be vaccinated and actually may not be, it is still sufficient to enforce the norm. This scenario seems plausible considering that the reported average vaccination rates were higher than those derived from administrative data ($\sim$73%^70^). Future studies could tackle this by gathering information directly from the contacts. Second, the data was sourced from a single country, which might not reflect the dynamics and patterns present in diverse cultural or socio-economic settings. Third, the survey was conducted at a specific point in the pandemic when the vaccine rollout was already nearing its peak. Future longitudinal studies in another pandemic context should aim at capturing the evolution of vaccination homophily dynamics over time. Finally, selection bias may also be caused by the vaccination status itself, whereby those who have been vaccinated seek to limit their physical contact to those who have also been vaccinated. In light of the aforementioned considerations, it is not possible to assert that vaccination homophily rates apply to the entire ego network, but rather to the individual’s face-to-face contact network solely.

Our study extended the understanding of vaccination homophily during the COVID-19 pandemic and the role of social networks in vaccination decisions. Tight-knit networks played an essential role in vaccination decisions. The strong clustering of unvaccinated individuals posed a great risk in preventing the spread of the disease which highlights the need for tailored, community-centred communication strategies during similar pandemics.

## Methods

### Data

The data analyzed in this manuscript was gathered within the framework of the MASZK study^69^. This comprehensive survey data collection initiative was conducted between April 2020 and June 2022, involving monthly cross-sectional samples ranging from 1,000 to 1,500 individuals. Respondents were selected through a multi-step, proportionally stratified, probabilistic sampling approach, ensuring a representative sample of the Hungarian adult population concerning gender, age, education, and domicile. Post-stratification weights were applied to correct for minor sampling errors. Data collection was performed with Computer-Assisted Telephone Interview (CATI) methodology. In the questionnaire, a core block was always included about aggregated contact patterns and symptoms of the respondents, as well as on their compliance with non-pharmaceutical interventions and social-demographic characteristics. Varying blocks were asked on current issues of the pandemic of the given month. The data under examination were collected in December 2021 and January 2022. The sample size was 1,000 in each month. In these months, the questionnaire included a network diary block, in which respondents reported on all contacts they had on the previous day and provided information on the contacts’ characteristics as well.

#### The contact diary approach

The most common measures of personal networks in surveys are based on one of the various network generators (e.g., name generator, position generator, or resource generator), which rely on the longer-term memory of the respondents and produce proxy measures of specific subsets of networks rather than actual and complete ones^81,82^. Thus, the resulting measures are exposed to several biases, e.g. a bias toward stronger ties^83^ or neglecting ties which are socially close but do not provide the examined resources^84^. Compared to the different generator methods, the contact diary approach has many notable positive features^68,71,81,85^. In this method, we ask the respondents to track and record all their interpersonal contacts (interactions) in a given period, and thus it can ’tap into the core element of egocentric networks more directly and more comprehensively’ (p. 172)^81^. The most basic approach of the contact diary registers the socio-demographic characteristics of the mentioned alters; however, more sophisticated and exhaustive information might also be gathered: e.g., the socioeconomic status of the alters, the circumstances of the interactions, or the characteristics of the relationship between ego and their alters^81^. Measuring daily contacts allows the researchers to collect information on ties of various strengths while avoiding biases towards either stronger or weaker ties^81,84^. Another strength of the diary approach is that it is ‘more familiar, natural, and unobtrusive to respondents’ (p. 196)^81^. Overall, while network generators provide proxy measures of a subset of the personal network and generally limit the number of alters to list, the contact diary approach aims to give information about a whole range of ties or ‘a complete profile of personal networks within a specific time period’ (p. 52) without restricting their number^81^. However, it is also worth noting that following the contact diary approach is very tedious and demanding, as it requires more time and more effort from both researchers and respondents^71,81,86^.

To utilize the originally demanding method in a nationally representative sample, the respondents were asked to list all personal interactions of the previous day, meaning that they had to record each interaction with the same person separately. The interactions that needed to be recorded were thoroughly defined: only face-to-face interactions lasting a few minutes or longer were recorded, excluding shorter encounters (e.g. greetings) and interactions over the Internet or telephone. The diary log consisted of three major parts: (1) The type and the place of the interaction, (2) the socio-demographic and other individual characteristics of the alter, and (3) the characteristics of the relationship between ego and alter. First, respondents had to name the contacted person. They could record the name, nickname, or initials of the alter. Then we asked the length and place of the interaction, as well as the circumstances of the encounter (e.g. whether there was physical contact, whether the parties were wearing masks), as the questionnaire was designed to isolate the types of contact that are relevant to the spreading of the COVID-19 virus. In the second part of the diary log, the characteristics of the alter were explored. Besides the traditional socio-demographic variables (gender, age, education), the respondent was also asked to rate the financial situation of the alter as well as whether the alter had received the COVID-19 vaccine. In the third part of the diary log, we asked questions about the relationship with the contacted person: the type of relationship (e.g., spouse, friend, coworker, etc.), whether they lived together in the same household, and how often they had met in the last six months.

### Measures

#### Dependent variable

The ego’s COVID-19 vaccination status was used as the dependent variable. Respondents were asked whether they had received at least one dose of the COVID-19 vaccine in a binary way (Yes/No).

#### Network-level predictors

Our key network-level predictor was the COVID-19 vaccination rate in the ego’s network. We only focused on contacts aged 18 or older because COVID-19 vaccines were only available for certain children aged 12 or older, and vaccination rates were very low among children at that time. Also, we believed that homophily is more relevant in adult-adult than in children-adult relationships. To calculate this rate we divided the number of vaccinated contacts by the number of the ego’s total contacts, thus 1 means a fully vaccinated network, whilst 0 means a fully unvaccinated network. This rate was not calculated if the ego did not have any contacts or could not provide any information about their contacts’ vaccination status (29.3%). The same calculation was performed for each sub-network of the ego (e.g., only for household members, only for friends, etc.). We provide descriptive statistics of respondents who provided contact information and those who have not (see Table S1). Those with no contact information are more likely to be females, to be aged above 60, have lower levels of education and have worse financial status. However, the two groups are not meaningfully different with regard to domicile and vaccination status. The strong differences observed along age are in line with other findings from Hungary^87^.

As the theory of social selection^1^ argues, people tend to surround themselves with similar others. To test whether the association between network vaccination rates and the ego’s vaccine uptake hold in different network compositions, we developed a set of other network-level control variables using the data gathered from the contact diaries. These measures covered the size of the network (number of contacts), the proportion of male contacts, contacts from different age and educational groups, kin- and strong-tie contacts and contacts from different sub-networks of the ego (household members, other relatives, friends and colleagues).

#### Ego-level predictors

The analysis comprised ego-level socio-demographic factors that prior research found to be related to vaccination decisions. Specifically gender, age, the highest level of education, type of settlement and income status of the respondent, as well as other variables measuring news consumption and the respondent’s experience with the COVID-19 virus. The highest completed education level was defined in four categories (primary education or less, lower secondary education, upper secondary education and tertiary education). The respondent’s place of residence was also categorized into four types: capital city (Budapest), county seat, other city, and village. The monthly net household income per capita provided by the respondent was used to determine their financial situation or the mean of the income categories provided if the respondent did not disclose the exact amount. The models also included variables on whether respondents have had a confirmed COVID-19 infection or knew of a friend or family member who became severely ill or possibly perished due to the virus. COVID-19-related news consumption was measured by asking respondents from which of the listed sources they gathered information about epidemic measures during the pandemic. A binary variable was used to measure selective exposure to the news posted on social media (1 if the respondent used only social media as a news source about COVID-19-related issues, and 0 in case of varied or no news consumption).

### Statistical approach

#### Vaccination homophily

To answer our first research question and describe the degree of vaccination homophily we first use descriptive statistics to summarize vaccination rates in different networks of both vaccinated and unvaccinated egos. Second, we calculate baseline and inbreeding homophily scores.

Baseline vaccination homophily (see e.g.,^73^), $H_{i}$ indicates the percentage of people within an individual’s network who have the same vaccination status as that individual. Let $s_{i}$ represent the average number of contacts individuals with a vaccination status of *i* have with others who share the same vaccination status. Meanwhile, let $d_{i}$ denote the average number of contacts that individuals with a vaccination status of *i* have with those of a different vaccination status. Baseline homophily index ($H_{i}$) is defined by

1 $$\begin{aligned} H_i = \frac{S_i}{S_i + d_i} \end{aligned}$$

This means the baseline homophily index can vary between 0 and 1, inclusive. If $H_{i}$ = 0, it indicates complete heterophily (all contacts are with those of different vaccination status), and if $H_{i}$ = 1, it indicates complete homophily (all contacts are with those of the same vaccination status). If $H_{i}$ is higher than $w_{i}$, which is the relative fraction of vaccination status of *i* in the population ($H_{i}$ >$w_{i}$) is suggestive of people leaning towards their own types. Nevertheless, when relatively large disproportionalities exist between groups in the population, the baseline homophily index is not considered an ideal measure of homophily because it does not entirely convey the extent of a group’s bias relative to its potential maximum bias. Coleman’s^72^ inbreeding homophily index ($IH_{i}$) takes into account these disproportionalities by normalizing the homophily index.

2 $$\begin{aligned} IH_i = \frac{H_i - w_i}{1 - w_i} \end{aligned}$$

This index effectively measures how much the observed homophily deviates from baseline homophily, scaled by the maximum potential bias (see the term 1 - $w_{i}$, where $w_{i}$ is the relative fraction of vaccination status *i* in the population). The inbreeding homophily index is negative in case of inbreeding heterophily and positive in case of inbreeding homophily (has the value of 1 in case of complete homophily), while 0 indicates that baseline homophily and inbreeding homophily are not different.

#### The role of the interpersonal networks in vaccine uptake

To examine the association between network vaccination patterns and the ego’s vaccine uptake, hierarchical logistic regression models were fitted. We selected logistic regression as the dependent variable, the ego’s vaccination status was binary (0, not vaccinated; 1, vaccinated). We note that, although our model specification implies the assumption that primarily the ego’s network influences the ego’s decision, and not vice versa, the direction of causality in this relationship is not clear-cut. There are compelling reasons to argue that egos can also exert influence on their social network, leading to a two-way interaction. Yet, building on social contagion theory, we opted for predicting the ego’s vaccination status and added multiple predictors for controlling for social selection theory. The reason for using hierarchical models is twofold. First, we wanted to capture the different explanatory power of network vaccination rates (Model 1), network characteristics (Model 2), and ego characteristics (Model 3). Second, we wanted to examine whether the effect of network vaccination rate changes when controlling for network- and ego characteristics. No imputation methods were used, those who did not report any contact in the last 24 hours or could not provide information about the vaccination status of any of their contacts were excluded from the analysis. Models were fitted using the *glm* function of the *stats* package (version 3.6.2) of R^88^. Instead of using raw estimates, we calculate Average Marginal Effects (AMEs) due to their interpretive advantage and their capacity to compare the impact of a predictor across different groups or different models^89^. The *margins* package^90^ of R was used to calculate AMEs.

We used the following regression models.

Model 1:

3 $$\begin{aligned} \log \left( \frac{p}{1 - p}\right) = \beta _0 + \beta _1\,network\,vaccination\,rate \end{aligned}$$

Model 2:

4 $$\begin{aligned} \begin{aligned}{}&\log \left( \frac{p}{1 - p}\right) \\&= \beta _0 + \beta _1\,network\,vaccination\,rate + \beta _2\,Number\,of\,contacts \\&+ \beta _3\,Rate\,of\,males\,in\,the\,network + \beta _4\,18--29\,years\,old\,rate \\&+ \beta _5\,30--39\,years\,old\,rate + \beta _6\,40--49\,years\,old\,rate \\&+ \beta _7\,50--59\,years\,old\,rate + \beta _8\,Lowest\,educational\,level\,rate \\&+ \beta _9\,Vocational\,school\,rate + \beta _{10}\,High\,school\,rate \\&+ \beta _{11}\,Lowest\,financial\,level\,rate + \beta _{12}\,Average\,financial\,level\,rate \\&+ \beta _{13}\,Kin\,rate + \beta _{14}{} \textit{Strong tie rate} + \beta _{15}\,Household\,member\,rate \\&+ \beta _{16}\,Other\,relative\,rate + \beta _{17}\,Friend\,rate + \beta _{18}\,Colleague\,rate \end{aligned} \end{aligned}$$

Model 3:

5 $$\begin{aligned} \begin{aligned}{}&\log \left( \frac{p}{1 - p}\right) \\&= \beta _0 + \beta _1\,network\,vaccination\,rate + \beta _2\,Number\,of\,contacts \\&+ \beta _3\,Rate\,of\,males\,in\,the\,network + \beta _4\,18--29\,years\,old\,rate \\&+ \beta _5\,30--39\,years\,old\,rate + \beta _6\,40--49\,years\,old\,rate \\&+ \beta _7\,50--59\,years\,old\,rate + \beta _8\,Lowest\,educational\,level\,rate \\&+ \beta _9\,Vocational\,school\,rate + \beta _{10}\,High\,school\,rate \\&+ \beta _{11}\,Lowest\,financial\,level\,rate + \beta _{12}\,Average\,financial\,level\,rate \\&+ \beta _{13}\,Kin\,rate + \beta _{14}{} \textit{Strong tie rate} + \beta _{15}\,Household\,member\,rate \\&+ \beta _{16}\,Other\,relative\,rate + \beta _{17}\,Friend\,rate + \beta _{18}\,Colleague\,rate \\&+ \beta _{19}\,Gender + \beta _{20}\,Age + \beta _{21}\,Education + \beta _{22}\,Settlement\,size \\&+ \beta _{23}\,Financial\,Situation + \beta _{24}\,Tested\,Positive + \beta _{25}\,Severe\,Contact\,with\,the\,Virus \\&+ \beta _{26}\,Selective\,Social\,Media\,Exposure + \beta _{27}\,Time\,of\,Data\,Collection \end{aligned} \end{aligned}$$

To discover whether the associations found in the models vary between different ego networks we fitted the same models for each network type. Consequently, this reduced our sample sizes and the statistical power of our analysis (N_household_ = 962, N_other relative_ = 399, N_friends_ = 239, N_colleagues_ = 324, N_kin_ = 1116, N_non-kin_ = 976, N_strong-tie_ = 1157, N_weak-tie_ = 762). Despite the reduction, the sample sizes remain substantial allowing for reliable conclusions. Again, AMEs are calculated because they allow for more precise comparisons.

### Supplementary Information

Supplementary Information.

## Data Availability

The data used in the analysis is available here: 10.17605/OSF.IO/STZRC.

## Code availability

The R code used in the analysis is available at the same storage: 10.17605/OSF.IO/STZRC.
