# Supplementary material for: Vaccination homophily in ego contact networks during the COVID-19 pandemic
Source: Sci Rep. 2024 Jul 5;14:15515. doi: 10.1038/s41598-024-65986-2 (PMC11226437; doi:10.1038/s41598-024-65986-2)
Supplement: Supplementary file 1 — Supplementary Information. [file 41598_2024_65986_MOESM1_ESM.pdf]

8 Supplementary information

Table S1. Descriptive statistics of those who provided valid contact information and those who have not

Variables	No contact information (N=586)	Valid contact information (N=1414)
Male	43.99	47.99
Female	56.01	52.01
Aged 18-29	11.70	19.72
30-39	12.45	18.70
40-49	15.81	19.85
50-59	15.18	15.00
Aged 60 or above	44.86	26.73
Lowest educational level	32.21	20.77
Vocational school	21.06	21.79
Secondary school	29.81	34.67
College	16.92	22.77
Small town	30.40	29.35
City	33.84	35.75
County seat	18.78	16.64
Capital	16.98	18.26
Poor financial situation	6.00	3.13
2	6.02	5.04
3	9.30	9.53
4	13.05	12.67
5	28.05	27.32
6	15.47	18.66
7	12.23	13.72
8	6.33	7.60
9	1.30	1.52
Good financial situation	2.27	0.81
Not vaccinated	16.90	17.07
Vaccinated	83.10	82.93

Table S2. Descriptive statistics of vaccination rates in different networks of the ego

Network type	N	Mean vacc. rate	SE	Median vacc. rate
Full Network	1469	83.38	0.8	100
Household	962	81.32	1.2	100
Other relatives	399	81.03	1.9	100
Friends	239	84.31	2.1	100
Colleagues	324	82.82	1.7	100
Kin	1116	81.11	1.1	100
Non-kin	976	84.83	0.9	100
Strong-tie	1157	81.69	1.0	100
Weak-tie	762	86.60	1.0	100

Table S3. Descriptive statistics of vaccination rates in different networks of vaccinated and unvaccinated egos

Network type	Vacc. status	N	Mean vacc. rate	SE	Median vacc. rate
Full Network	Unvacc.	255	48.69	2.5	50
	Vacc.	1214	90.52	0.7	100
Household	Unvacc.	174	30.53	3.4	0
	Vacc.	788	92.24	0.9	100
Other relatives	Unvacc.	70	47.42	5.4	50
	Vacc.	329	88.02	1.7	100
Friends	Unvacc.	47	67.31	5.9	100
	Vacc.	192	88.35	2.1	100
Colleagues	Unvacc.	67	57.16	4.9	60
	Vacc.	257	89.33	1.4	100
Kin	Unvacc.	202	34.57	3.1	0
	Vacc.	914	91.16	0.9	100
Non-kin	Unvacc.	174	65.06	2.9	75
	Vacc.	802	89.03	0.9	100
Strong-tie	Unvacc.	205	37.36	3.0	0
	Vacc.	952	91.02	0.8	100
Weak-tie	Unvacc.	131	69.31	3.3	100
	Vacc.	631	90.12	1.0	100

Table S4. Results of the models fitted on vaccine uptake of the respondent

	Model 1			Model 2			Model 3		
	AME	SE	*p*	AME	SE	*p*	AME	SE	*p*
Network vaccination rate	0.36	0.02	<0.001	0.35	0.02	<0.001	0.32	0.02	<0.001
Network characteristics									
# of Contacts				0.00	0.00	.467	0.00	0.00	.557
Rate of males in the network				0.01	0.03	.568	0.01	0.03	.783
18-29 years old rate				-0.05	0.04	.136	0.03	0.04	.475
30-39 years old rate				-0.02	0.04	.585	0.03	0.04	.492
40-49 years old rate				-0.05	0.04	.138	-0.02	0.04	.579
50-59 years old rate				-0.05	0.04	.167	-0.01	0.04	.746
Lowest educational level rate				-0.09	0.04	.026	-0.07	0.05	.135
Vocational school rate				-0.10	0.03	.003	-0.09	0.04	.016
High school rate				-0.09	0.03	.010	-0.08	0.03	.023
Lowest financial level rate				-0.02	0.04	.635	0.01	0.04	.891
Average financial level rate				0.01	0.03	.773	0.03	0.03	.447
Kin rate				0.08	0.05	.111	0.06	0.05	.223
Strong tie rate				0.01	0.03	.637	0.02	0.03	.442
Household member rate				-0.09	0.06	.123	-0.06	0.05	.236
Other relative rate				-0.06	0.06	.371	-0.04	0.06	.504
Friend rate				-0.04	0.04	.391	-0.01	0.04	.787
Colleague rate				-0.03	0.04	.388	-0.02	0.04	.598
Ego characteristics									
Gender (ref.: Male)							0.03	0.02	0.09
Age							0.00	0.00	<0.001
Education							0.01	0.01	.484
Other city (Ref.: Small town)							0.03	0.02	.159
County seat							0.02	0.03	.549
Capital							0.08	0.03	.002
Financial Situation							0.01	0.01	.032
Tested Positive							-0.02	0.02	.224
Severe Contact with the Virus							0.04	0.02	.017
Selective Social Media Exposure							-0.01	0.02	.587
Time of Data Collection (ref.: 11/21)							-0.06	0.03	.048
N		1458			1458			1458	
Pseudo R^2		0.25			0.27			0.30	

Table S5. Results of the models fitted on vaccine uptake of the ego among different networks of the respondent

	Household			Other relatives			Friends			Colleagues		
	AME	SE	*p*	AME	SE	*p*	AME	SE	*p*	AME	SE	*p*
Vaccination rate in the network	0.31	0.01	<0.001	0.24	0.03	<0.001	0.15	0.06	0.018	0.32	0.05	<0.001
Network characteristics												
# of Contacts	0.00	0.00	0.975	0.01	0.01	0.343	0.00	0.01	0.885	0.00	0.00	0.264
Rate of males in the network	0.01	0.03	0.853	-0.01	0.06	0.862	0.02	0.09	0.788	-0.03	0.08	0.76
18-29 years old rate	0.1	0.05	0.035	-0.03	0.08	0.738	-0.12	0.13	0.341	0.2	0.13	0.122
30-39 years old rate	0.07	0.04	0.107	-0.05	0.08	0.545	-0.1	0.15	0.503	0.05	0.12	0.684
40-49 years old rate	0.03	0.04	0.451	-0.09	0.08	0.253	-0.14	0.13	0.295	0.07	0.12	0.578
50-59 years old rate	0.02	0.04	0.691	-0.03	0.08	0.708	0.02	0.15	0.913	0.31	0.16	0.047
Lowest educational level rate	-0.03	0.05	0.596	-0.08	0.12	0.518	-0.22	0.16	0.155	-0.19	0.13	0.157
Vocational school rate	-0.07	0.04	0.094	-0.15	0.09	0.106	-0.33	0.13	0.008	-0.2	0.09	0.019
High school rate	-0.07	0.04	0.074	-0.18	0.09	0.058	-0.26	0.12	0.027	-0.16	0.08	0.054
Lowest financial level rate	0.01	0.05	0.853	-0.03	0.1	0.742	-0.11	0.14	0.445	0.06	0.1	0.578
Average financial level rate	0.05	0.04	0.185	-0.04	0.09	0.666	-0.04	0.1	0.683	0.08	0.09	0.357
Kin rate	0.09	0.05	0.082	0.08	0.13	0.513	0.31	0.31	0.328	0.09	0.28	0.745
Strong tie rate	0.03	0.03	0.452	0.01	0.05	0.784	0.15	0.09	0.071	-0.01	0.05	0.898
Household member rate	-0.15	0.07	0.033	0.04	0.16	0.789	-0.49	0.32	0.134	-0.06	0.28	0.838
Other relative rate	-0.1	0.08	0.215	0	0.16	0.984	-0.37	0.37	0.311	-0.4	0.32	0.211
Friend rate	-0.03	0.08	0.714	0.12	0.12	0.347	-0.06	0.15	0.694	0.12	0.22	0.596
Colleague rate	-0.1	0.06	0.095	-0.05	0.12	0.7	-0.18	0.18	0.329	0.17	0.12	0.143
Ego characteristics												
Gender (ref.: Male)	0.04	0.02	0.064	0.00	0.04	0.929	-0.02	0.05	0.642	0.08	0.05	0.086
Age	0.00	0.00	<0.001	0.01	0.00	<0.001	0.00	0.00	0.883	0.00	0.00	0.011
Education	0.01	0.01	0.666	0.02	0.02	0.418	0.03	0.03	0.42	-0.03	0.03	0.266
Other city (Ref.: Small town)	0.04	0.02	0.083	-0.04	0.04	0.386	-0.03	0.06	0.642	0.16	0.06	0.005
County seat	0.02	0.03	0.407	0	0.05	0.981	0.04	0.07	0.573	0.2	0.06	0.002
Capital	0.09	0.03	0.005	0.08	0.05	0.146	0.03	0.08	0.663	0.2	0.07	0.007
Financial Situation	0.01	0.01	0.178	0.01	0.01	0.19	-0.02	0.02	0.297	0.03	0.01	0.026
Tested Positive	-0.02	0.02	0.247	0.01	0.04	0.811	0.02	0.06	0.715	-0.01	0.04	0.826
Severe Contact with the Virus	0.06	0.02	0.004	0.00	0.04	0.935	0.06	0.05	0.295	0.11	0.04	0.007
Selective Social Media Exposure	-0.02	0.04	0.519	-0.04	0.07	0.558	-0.2	0.08	0.013	-0.07	0.08	0.377
Time of Data Collection (ref.: 11/21)	0.01	0.02	0.742	-0.01	0.04	0.73	-0.06	0.05	0.253	-0.07	0.04	0.074
N		960			397			236			323	
Pseudo R^2		0.45			0.31			0.23			0.34	

Table S6. Results of the models fitted on vaccine uptake of the ego among different networks of the ego cont.

	Kin			Non-kin			Strong-tie			Weak-tie		
	AME	SE	*p*	AME	SE	*p*	AME	SE	*p*	AME	SE	*p*
Vaccination rate in the network	0.31	0.01	<0.001	0.21	0.03	<0.001	0.32	0.01	<0.001	0.17	0.03	<0.001
Network characteristics												
# of Contacts	0.00	0.00	0.384	0.00	0.00	0.915	0.00	0.00	0.469	0.00	0.00	0.309
Rate of males in the network	0.00	0.03	0.944	0.00	0.04	0.914	0.03	0.03	0.324	-0.04	0.04	0.33
18-29 years old rate	0.06	0.04	0.13	0.00	0.06	0.976	0.08	0.04	0.06	0.00	0.07	0.944
30-39 years old rate	0.07	0.04	0.091	-0.01	0.06	0.826	0.06	0.04	0.16	-0.07	0.07	0.295
40-49 years old rate	0.02	0.04	0.645	-0.06	0.05	0.275	0.03	0.04	0.462	-0.06	0.06	0.339
50-59 years old rate	0.02	0.04	0.67	-0.01	0.06	0.823	0.04	0.04	0.363	-0.05	0.06	0.419
Lowest educational level rate	0.00	0.05	0.949	-0.17	0.07	0.013	-0.06	0.05	0.197	-0.18	0.08	0.025
Vocational school rate	-0.07	0.04	0.102	-0.21	0.05	0.00	-0.08	0.04	0.029	-0.22	0.06	<0.001
High school rate	-0.07	0.04	0.055	-0.14	0.05	0.007	-0.06	0.04	0.086	-0.19	0.06	0.002
Lowest financial level rate	0.02	0.05	0.74	-0.02	0.06	0.701	0.00	0.04	0.981	-0.07	0.07	0.334
Average financial level rate	0.05	0.04	0.202	-0.02	0.05	0.635	0.04	0.04	0.28	-0.05	0.06	0.441
Kin rate	0.05	0.06	0.403	0.12	0.08	0.147	0.1	0.05	0.062	0.2	0.1	0.045
Strong tie rate	0.01	0.03	0.682	0.01	0.03	0.662	0.00	0.03	0.902	0.00	0.05	0.994
Household member rate	-0.06	0.07	0.434	-0.14	0.08	0.089	-0.09	0.06	0.121	-0.26	0.11	0.015
Other relative rate	-0.05	0.08	0.503	-0.14	0.09	0.15	-0.08	0.07	0.237	-0.17	0.11	0.102
Friend rate	0.02	0.07	0.808	-0.03	0.05	0.58	0.00	0.06	0.961	-0.05	0.05	0.374
Colleague rate	-0.07	0.06	0.228	-0.02	0.04	0.553	-0.04	0.04	0.309	0.01	0.05	0.817
Ego characteristics												
Gender (ref.: Male)	0.03	0.02	0.19	0.02	0.02	0.325	0.03	0.02	0.062	0.01	0.03	0.668
Age	0.00	0.00	<0.001	0.00	0.00	<0.001	0.00	0.00	<0.001	0.00	0.00	<0.001
Education	0.01	0.01	0.268	0.00	0.02	0.977	0.00	0.01	0.815	0.03	0.02	0.162
Other city (Ref.: Small town)	0.02	0.02	0.425	0.03	0.03	0.296	0.03	0.02	0.232	0.03	0.03	0.367
County seat	0.03	0.03	0.296	0.02	0.04	0.558	0.03	0.03	0.289	-0.03	0.04	0.531
Capital	0.08	0.03	0.013	0.11	0.03	0.001	0.09	0.03	0.003	0.1	0.04	0.009
Financial Situation	0.01	0.01	0.219	0.02	0.01	0.006	0.01	0.01	0.252	0.02	0.01	0.056
Tested Positive	-0.02	0.02	0.315	-0.05	0.03	0.08	-0.02	0.02	0.253	-0.03	0.03	0.384
Severe Contact with the Virus	0.04	0.02	0.051	0.06	0.02	0.011	0.06	0.02	0.004	0.04	0.03	0.125
Selective Social Media Exposure	-0.03	0.04	0.503	-0.09	0.05	0.043	-0.03	0.04	0.411	-0.09	0.05	0.079
Time of Data Collection (ref.: 11/21)	0.00	0.02	0.957	-0.04	0.02	0.104	0.01	0.02	0.533	-0.06	0.03	0.035
N		1113			966			1154			753	
Pseudo R^2		0.40			0.18			0.39			0.18	
